# Perceptions of Self-Testing for Chlamydia: Understanding and Predicting Self-Test Use

**DOI:** 10.3390/healthcare4020025

**Published:** 2016-05-10

**Authors:** Rachael Powell, Helen M. Pattison, John F. Marriott

**Affiliations:** 1 School of Psychological Sciences & Manchester Centre for Health Psychology, University of Manchester, Manchester M13 9PL, United Kingdom; 2 School of Life and Health Sciences, Aston University, Birmingham B4 7ET, United Kingdom; h.m.pattison@aston.ac.uk; 3 School of Pharmacy, College of Medical and Dental Sciences, University of Birmingham; Birmingham B15 2TT, United Kingdom; J.F.Marriott@bham.ac.uk

**Keywords:** chlamydia, self-testing, Protection Motivation Theory, Theory of Planned Behaviour, home testing

## Abstract

*Background*: Self-testing technology allows people to test themselves for chlamydia without professional support. This may result in reassurance and wider access to chlamydia testing, but anxiety could occur on receipt of positive results. This study aimed to identify factors important in understanding self-testing for chlamydia outside formal screening contexts, to explore the potential impacts of self-testing on individuals, and to identify theoretical constructs to form a Framework for future research and intervention development. *Methods*: Eighteen university students participated in semi-structured interviews; eleven had self-tested for chlamydia. Data were analysed thematically using a Framework approach. *Results*: Perceived benefits of self-testing included its being convenient, anonymous and not requiring physical examination. There was concern about test accuracy and some participants lacked confidence in using vulvo-vaginal swabs. While some participants expressed concern about the absence of professional support, all said they would seek help on receiving a positive result. Factors identified in Protection Motivation Theory and the Theory of Planned Behaviour, such as response efficacy and self-efficacy, were found to be highly salient to participants in thinking about self-testing. *Conclusions*: These exploratory findings suggest that self-testing independently of formal health care systems may no more negatively impact people than being tested by health care professionals. Participants’ perceptions about self-testing behaviour were consistent with psychological theories. Findings suggest that interventions which increase confidence in using self-tests and that provide reassurance of test accuracy may increase self-test intentions.

## 1. Introduction

Self-test technology allows people to test themselves for illness without intervention from health care professionals. Self-testing could benefit users, facilitating testing and treatment of people who might not otherwise access medical care. Sexually transmitted infections (STIs) such as chlamydia are associated with high levels of stigma, so facilitating self-test use could increase diagnosis amongst people who would not otherwise come forward for testing. In a UK genito-urinary medical clinic setting, self-sampling (providing urine (men) or vaginal swab samples (women)) was shown to be an acceptable alternative to physical examination in a randomized controlled trial with 391 participants [1]. Xu *et al.* (2011) found that women sent self-sampling kits for chlamydia were more likely to be re-screened than those invited to attend a clinic [2]. These researchers conducted two randomized controlled trials including women who had been treated for chlamydia infection either in sexually transmitted disease (STD) clinics (n = 880) or family planning clinics (n = 412) in cities in Missouri and Mississippi, USA. Participants were scheduled to be rescreened for chlamydia three months after initial treatment and, in both settings, were randomly assigned to either being given an appointment to return to the clinic or to receive a vaginal swab self-collection kit sent to their home. In the STD clinic sample, 26.7% of women who were sent home sampling kits re-screened, compared with 19.1% of those invited to attend the clinic (*p* = 0.01); in the family planning clinic sample, 40.8% of those sent home kits re-screened compared with 20.7% invited to attend the clinic (*p* < 0.001) [2]. However, both of these studies depended on individuals first attending a clinic. Research is, therefore, needed to consider the perceptions of people who test in non-medical settings and who receive results without medical support. In addition, there are concerns that self-testing, or receiving positive results through self-testing, could cause anxiety in the absence of professional support [3].

Genital chlamydia (*Chlamydia trachomatis*) is the most commonly reported STI in Europe and USA [4,5,6]. However, if detected, the disease can be effectively treated [7]. Since 2003, the National Chlamydia Screening Programme (NCSP) in England has been implemented for under-25 year-olds [8]. The programme uses strategies including distributing self-tests from GP surgeries, pharmacies and non-medical settings, e.g., by post and at university campuses. Self-tests are also available independently of the NCSP, purchased from pharmacies, and on the internet. Tests usually require people to send urine samples or vulvo-vaginal swabs to a laboratory for testing, with results being communicated by text or email rather than in person by a health care professional. Some tests allow people to test their own sample at home.

The impact of inviting people to screen for chlamydia using postal self-test kits was examined in a large scale UK study [9]. Invitations to submit home-collected urine samples (and vulvo-vaginal swabs for women) were sent to 19,773 adults aged 16–39 years. Participants completed questionnaires at three time points: one month before kits were sent, when kits were received, and after receiving a negative result. Screening did not appear to lead to increased anxiety. For men, anxiety was lower on receiving the kit than at one month previously; for women, anxiety was lower after receiving negative results. A qualitative study within this project interviewed participants with positive and negative results [10,11]. Minor anxiety was reported while waiting for test results, and some reported concern about test accuracy, but the most severe anxiety was experienced on receiving positive test results. Participants were informed of positive results by a nurse; learning of a positive status without professional support could have a greater impact on anxiety.

For chlamydia transmission to fall in young adults, testing rates need to be maintained at above 35% [12]. In 2014, testing rates of 15–24 year olds in England ranged from 21% to 28% by region [13]. Identifying factors that predict self-testing could allow the development of effective interventions to increase testing behaviour outside traditional medical settings. As argued by Michie *et al.*, it is important to use theoretical models because “interventions are likely to be more effective if they target causal determinants of behaviour and behaviour change” [14]. Little research in the area of chlamydia testing has incorporated theory into study design or data interpretation. Two models that have been used in predicting behaviour in other screening contexts are Protection Motivation Theory [15,16] and the Theory of Planned Behaviour [17].

The main aim of the present study was to explore self-testing for chlamydia from the perspective of young adults, to identify factors that may predict self-testing outside the context of formal screening programmes and to understand how self-test use impacts on individuals. However, a key secondary aim was to identify theoretical domains that explain the qualitative findings and which could form an effective framework for further research. 

## 2. Materials and Methods 

Ethical approval was received from the University Ethics Committee.

### 2.1. Participants

Participants were students at a university in the West Midlands, UK, and were recruited using emails to student mailing lists. Emails stated that we would particularly like to talk with people who had used a self-test kit for chlamydia, or whose partner had used one. Participants were paid £20. Nineteen people participated in an interview; one had never been sexually active (and had not self-tested) and was excluded from analysis. 

### 2.2. Procedure

Semi-structured interviews were conducted by the first author at the university, audio-recorded and transcribed verbatim. Topics covered included experiences of self-testing, perceived advantages and disadvantages of self-testing, how participants would feel on receiving a positive result and others’ perceptions about participants self-testing for chlamydia. A funnelling approach was used: earlier questions were broad to encourage participants to discuss aspects they considered important; later items were more focused, ensuring that aspects relevant to theories were discussed. Interviews were audio-recorded and transcribed verbatim. Participants were recruited until data saturation was observed *i.e*., no new themes were identified, and there were no issues arising regarding categorising data [18]. When it became clear to the lead and co-author, through detailed discussion of the data, that no new issues were arising in interviews, recruitment ceased.

### 2.3. Analysis

A thematic analysis of the interviews was conducted using a Framework approach [19,20]. The analysis was thematic in that we aimed to organize, describe, and understand the thoughts, feelings, and experiences of participants related to self-testing for chlamydia. A Framework approach [20] was followed to conduct the analysis because it is a systematic approach which allows comparisons to be made both within and between participants, and it can be easily accessed by other people: there is a clear trail by which other researchers can see the steps made by the analyst and assess the validity of the analysis. Transcripts were read and re-read, and thoughts, comments, and themes were noted on the manuscript. A list of superordinate and sub-themes was devised and used to code the manuscript line-by-line. The coded manuscripts enabled the creation of charts indexing extracts belonging to each theme for each participant, with details of the location of extracts in interview transcripts. These charts allowed the researchers to view the content of themes within and between participants, without losing sight of how extracts were embedded in the data. The charts were used to structure the study results according to theme with supporting evidence given verbatim. The lead author met with the second author regularly throughout the project to discuss progress and issues arising, and to make the decision as to when to stop interviewing. The detailed analysis records made by the lead author allowed a co-author to undertake independent analysis of a sample of transcripts (4 of 18, 22%). The co-author agreed with the understanding of the data reached by the lead author, ensuring validity of the analysis. After 18 interviews were complete the lead and second author decided no further new data were arising and saturation point had been met. The analysis was primarily structured around the participants’ responses rather than by the theoretical models. To meet the secondary aim of the study, the qualitative findings were then related to theoretical models that have been used to explain and predict behaviour in other screening contexts using abductive inference.

## 3. Results

### 3.1. Description of Participants

The participants are described in Table 1. When referring to participants in-text, “F” denotes female; “M” denotes male; “ST” indicates that the participant reported having self-tested; “NT” indicates that the participant had not self-tested. Participants are numbered from 1 to 19; participant number 8 was excluded from analysis because they had not been sexually active, leaving the sample of 18 participants. All participants who had self-tested received negative test results; one participant (F2(ST)) was awaiting her result. Interviews ranged from 25 to 48 minutes in length; the median duration was 35 minutes.

### 3.2. Experiences of Using Tests

Of the three participants who had self-tested at home, two were invited to screen for chlamydia by post through local National Health Service initiatives (F5(ST), F19(ST)). The third (F15(ST)) picked up a kit from her general practitioner’s (GP) surgery. In discussing reasons for self-testing the concept of “ease” was raised by each of these participants, but with different meanings implied. “Easy” was used to indicate the test not being difficult to perform—“it’s free, it’s easy so you know, I’ve got nothing to lose” (F5(ST)), being less intimidating than clinic testing—“I wouldn’t have actually plucked up the courage to go to a clinic…it just seemed really easy” (F19(ST)), and being convenient and private—“easier to do in my own time, nobody else has to be involved” (F15(ST)). However, F19(ST) found the process of self-testing worrying, particularly because she had expected to receive a urine test but was instead sent a vulvo-vaginal swab test which she did not feel confident with using:

“because it really wasn’t what I expected, um, I just expected to do a urine test and it wasn’t and it gave you sort of an instruction list of how to do the swab em, but yeah it was a bit scary I first thought... I sort of thought what if I do something wrong and also in the letter it said some results can be inconclusive and I just thought that will be me and, and they’ll send me a text or a letter saying it didn’t work and I’d have to do it again, so I think that was my first worry that it wouldn’t work and that I was doing it wrong”.

Self-test use was opportunistic for these participants—“I wouldn’t have even thought to do one if I hadn’t seen them” (F15(ST)). However, they welcomed the opportunity to gain reassurance, knowing that chlamydia can be symptomless—“you don’t show anything” (F5(ST)).

Five participants self-tested through the NCSP at the Students’ Union, with free t-shirts as a reward. This seemed an effective incentive: participants gave the t-shirt as their key motivator in doing the test, although convenience was also mentioned. For F17(ST), social influence was important—both she and her friend decided to take part, there was “a bit of a buzz”, and she wished to support the staff member—“I felt a bit sorry for the guy that asked”—but she also welcomed the reassurance of a negative result. It may be that the testing environment allowed people who were actually concerned about chlamydia to test without stigma; it may be easier to tell friends and a researcher that they tested for a t-shirt than to admit to worries about having an STI:

“I was with her I was like oh do you wanna do it and she was like er, yeah, okay, cos I didn’t want to look like oh I’ve already got an STI so I think I’d better do it, so I was sort of, you know, waiting for her to offer a bit of encouragement”

(F17(ST))

Three participants self-tested within health clinics, while attending the clinic for other reasons. Testing again seemed opportunistic “as they offered I didn’t see the point in turning it down” (F2(ST)). Being able to take her own sample, independently of a professional, was regarded as a benefit by F18(ST)—“I’d rather do it myself…definitely, a hundred percent” (F18(ST)). In contrast, F6(ST) only self-tested because she did not perceive herself to have a choice:

F6(ST): they said “here you go, do it yourself”

Interviewer: “If you’d had the choice before you went for it, what would you have opted for do you think”?

F6(ST) “I would have probably asked them to do it in all honesty; I probably would have asked them, yeah” (and later) “I didn’t necessarily have the confidence in myself”

This participant (F6(ST)) was uncomfortable about self-testing because she lacked confidence in her ability to correctly perform the procedure (taking a vulvo-vaginal swab sample). Having the option of having a urine test rather than using a vaginal swab would have made her more comfortable with the process—“you can’t really go wrong”.

Most participants seemed not to be anxious while waiting for results—“I’d kind of forgotten about it” (F18(ST)) although anxiety could result—“between taking the test and getting the results I’d got more sort of anxious” (F19(ST)). In this sample, participants apparently self-tested because they were given the opportunity to test; awaiting test results might be more anxiety-provoking for people who actively seek testing. On receiving negative results, participants widely reported relief suggesting that the possibility of chlamydia was still worrying, even though they did not perceive themselves to be sufficiently at risk to seek out tests:

“I just kept thinking about it every day um, because I didn’t have any reason to be concerned about having Chlamydia, I just wanted it to be for peace of mind”

(F19(ST))

### 3.3. Barriers to Self-Testing

#### 3.3.1. Method of Testing

The technology itself was important in shaping views about self-testing. Female participants mostly used vulvo-vaginal swabs, although it is not clear why this is—whether out of choice, or whether this was the default type of kit offered to them. For example, as noted earlier, F19(ST) had expected to receive a urine test but instead was sent a vulvo-vaginal swab kit and F6(ST) did not feel that she was given a choice. F17(ST) mentioned having used a vulvo-vaginal swab because she had not realised she could have provided a urine sample:

F17(ST): “I didn’t realise that you had to, that you could like pee on it as well”;

Interviewer: “Okay”

F17(ST): “Someone told me and my friend came back with one this year, like just a few months ago, and I said oh yeah but that’s the girl’s one, boys can’t use that because girls do a different method and she was like oh no, everyone can just pee on it if you want. I probably would have preferred doing that thinking about it now but I just didn’t realise”.

While some found vulvo-vaginal swabs straightforward to use, others found them less so—“I didn’t necessarily have the confidence in myself” (F6(ST)) and the prospect of a vulvo-vaginal swab could be a barrier to self-testing for some individuals—“I wouldn’t be able to do it on myself” (F13(NT)). 

#### 3.3.2. Cost

Cost was important in deciding whether or not to self-test: F1(NT) gave cost as the most important reason for going to a clinic, where testing would be free in the UK, rather than self-testing (even though tests provided through the UK’s National Health Service or NCSP are also free for this age group). Even though students have limited financial resources and cost was an issue for them, concern about the quality of a cheap test was raised—“it might not be as accurate” (F12(NT)). Thus, the relationship between self-testing and cost is complex: cost can be prohibitive, but also indicative of quality and test reliability.

#### 3.3.3. Concerns about Test Accuracy

A key worry about self-testing was concern about test accuracy, and whether errors in sampling could cause false negative results. The dangers of a false negative result were articulated by F7(NT): “more serious than not doing it at all”, as people would not then seek treatment and could unwittingly infect others. Participants were also concerned about the risk of discovery, in particular when acquiring the self-test—“I would have to go somewhere where I didn’t know anyone” (F4(NT)) and on receiving results. Participants also raised concerns about accidents in posting samples, e.g., sample containers breaking, being lost, or results being mixed up.

#### 3.3.4. Absence of Professional Support

Finally, the absence of professional support was raised as a disadvantage. Some participants were more confident in a professional taking a sample than in themselves self-sampling, and receiving results from a professional was seen as advantageous for receiving reassurance and identifying necessary action—“with a text message you would probably be a bit more like ‘hah’ (breath intake)…because then you’d have to…make another appointment” (F18(ST)). 

### 3.4. Facilitators to Self-Testing

#### 3.4.1. Privacy

Most participants mentioned privacy or anonymity as a benefit to self-testing, with the alternative methods of testing (seeing a GP or going to a specialist clinic) being seen as embarrassing. This anticipated embarrassment could be caused by the thoughts of discussing a taboo topic—sex and sexually transmitted infection, e.g.,:

“If you are someone who is quite embarrassed about going to the doctor for that sort of thing then it’s handy for that because you just, it’s all, you don’t have to come face-to-face with anyone. Like I didn’t even have to ask someone to pick it up it was just on the side so it’s useful for that”

(F15(ST))

Some participants were more explicit, worrying about others judging them for their sexual behaviour, and the consequences of this if they were to go to a clinic:

“I’d find it quite embarrassing going to a clinic and just like you know, everyone knowing you had unprotected sex or whatever. But erm, yes so I think the idea of doing it at home is like, is quite a good thing”

(F4(NT))

“it’s confidential, you don’t have to worry about what your doctor might think of you, worry what anyone else might say to you”

(F18(ST))

#### 3.4.2. Absence of Physical Examination

Avoiding a physical examination was clearly important to some participants—“other people have taken swabs and that is just horrible” (F18(ST)). As people find being tested by a professional to be aversive, the possibility that self-testing could enable some people to test who would not otherwise be tested was noted by participants—“I’ve another friend who’s never had an STI check ever…she said ‘oh no, I won’t, I won’t, I can’t, I can’t’. So I think for people like that it’s a really good idea” (F1(NT)). 

#### 3.4.3. Convenience

Participants viewed self-testing as more convenient than visiting a clinic or making a doctor’s appointment:

“I think cos its quite convenient as well cos I think if you’re working and everything it is a bit of a hassle trying to, it’s a hassle for me just to try and get to see my doctor; you have to kind of phone in advance and you have to phone in at a certain time and it’s a bit annoying. And then you have to have you’ve got the waiting as well. So I think it’s just, it’s more convenient and you can do it whenever really”

(F4(NT))

“I think it’s a great idea that you can have them in your own home to save you having to queue up for appointments etcetera, etcetera and they can take quite a while”

(F6(ST))

These quotes illustrate how convenience was particularly considered in the context of perceived waiting time that could be needed to have a medical appointment, and the challenges of fitting an appointment in alongside other life commitments.

#### 3.4.4. Control

Some participants seemed to appreciate having control over when, where, and how they could carry out a self-test, as opposed to the rigidity of having a clinic appointment:

“it’s something you work up to, towards as well so it’s like once you’ve decided yeah, I’m going to do it kind of thing, you can do it whenever you feel like you know most bravest”

(F4(NT))

“you can choose as well the method… but you feel like less pressured and more relaxed and you can take your time and you still have the option to send it even if you’ve done it or not. Um, I don’t know it’s just; it’s like, it’s all your own decision”

(F5(ST))

Having a sense of control over the process appeared to be reassuring for participants, ensuring that an unpleasant process would be carried out at a time, in a way, and at a speed that was comfortable for them.

When considering both benefits and costs to using a self-test kit, it was clear that participants did not consider their options as either self-testing or not testing at all. Instead, participants considered self-testing in the context of another behaviour: going to a clinic to be tested.

### 3.5. Anticipated Responses to Positive Test Results

No one in our sample reported having been given a positive test result for chlamydia. They were asked how they would hypothetically respond to this scenario. Participants anticipated a range of negative emotions if they were to receive a positive result including “scared and upset” (F5(ST)), “I’d feel anxious, worried” (M9(ST)), “I think I’d be a little bit devastated” (F11(NT)), and “I probably would have been quite shocked” (F17(ST)). 

For some, a hypothetical positive result would be worrying as a result of learning of a health threat and because of the unknown aspects of an infection—“scared because I don’t really know…how treatable it is” (F12(NT)); knowing chlamydia to be treatable led to lower anticipated anxiety—“I’d have probably been a bit worried but I know that chlamydia can be cleared up pretty easily” (F18(ST)). 

For others, anticipated negative emotion resulted from how they believed they would have become infected. For example, F12(NT) seemed to take responsibility for her hypothetical infection, and anticipated being disappointed in herself—“I think I’d panic. And I’d probably be kicking myself, I should have been more careful”. Others felt that, in the event of an infection, their partners would be responsible and an infection would indicate infidelity. A positive result would, therefore, have implications for their relationship, causing concern—“I would be worried how I caught it” and anger—“He may get a punch in the face” (F2(ST)).

The lack of professional support in the event of a positive self-test result seemed to increase anticipated anxiety for some participants:

“if you actually test positive and you have got the STD then I think I would be a bit worried, I wouldn’t know like what to do next”

(F4(NT))

“I’d be a little scared because that’s the thing, I need my doctor to just tell me, calm me down and tell me like, you know, it’s not the end of the world we can fix it. But if I’m at home by myself, you know. I think I would just go a little crazy because I wouldn’t know what to do with it”

(F13(NT))

However, for others, the absence of immediate medical support was not considered problematic—“I don’t think that that would be any different had I gone to the GP and they told me the same thing” (F7(NT)) and, despite thinking that she would be worried if she tested positive with a self-test (as noted above), F4(NT) still felt that being able to self-test was helpful:

“I think it’s actually quite a good idea because I think it’s quite, I’d find it quite embarrassing going to a clinic and just like you know, everyone knowing you had unprotected sex or whatever. But erm, yes so I think the idea of doing it at home is like, is quite a good thing”.

Thus, concern about lack of support in the event of a positive test may not necessarily deter people from using self-tests. 

Thoughts about long term consequences (*i.e*., infertility) could be important in either increasing or reducing concern:

“I don’t think I want kids…so it’s not like I’d be that devastated if something bad had happened. But…in the future if I changed my mind…that might be worrying” (F18(ST)). Positive emotional impact on receiving a positive hypothetical diagnosis was also considered: reassurance on identifying the disease and being able to access treatment—“I think I’d be reassured in some senses because I’d know that ok, that’s what it is” (F7(NT)); “I’d have been really glad that I did the test” (F5(ST)). 

Concern was voiced that some people might “freak out” on receiving a positive result and not seek medical care (F1(NT)). However, all our participants said they themselves would seek medical care, often as a matter of urgency, in the event of a positive result. 

### 3.6. Social Perceptions 

Expectations and experiences of partners’ responses to participants self-testing depended on the context of test use, with why someone would want to test causing concern rather than the self-testing, itself. For some, whether or not the disease was present was important—“he was like ‘oh, that’s good’” (F17(ST)). For others, the context of testing was crucial with *why* someone would need to be tested causing concern. Some thought partners would see self-testing as implying promiscuity or unsafe practice before the present relationship, or unfaithfulness during the relationship. Concerns about a negative reaction could lead to secrecy about self-testing—“he would’ve sort of asked me if I’d sort of been with someone else so I just thought it was best to keep it from him” (F19(ST)). A partner finding out about testing could have particularly negative consequences—“I think they’d probably break up with me” (M9(ST)). Thus, reservations resulted not from self-testing itself, but the implications of seeming at risk.

Similar issues were raised when participants were asked how they would feel about their partners self-testing. It was not self-testing that caused concern, but why they wanted to test—“I’d probably think why would they have it...I’d also panic the fact that I may have it” (F12(NT)); “I’d just like to know whether they thought they might have been infected of whether they did it just for the hell of it” (F10(ST)). F10(ST) had used a test at the Student’s Union, where everyone was being encouraged to test, so the implications of a partner wanting to self-test may have seemed less serious than for someone who tested by buying their own kit for home use. Two participants (both male) acknowledged that knowing their partner had self-tested could affect how they viewed them and how attractive they found them—“It might change the way I thought about them slightly… there’s nothing very sexy about a sexually transmitted disease” (M16(ST)). M9(ST) had particularly negative perceptions of a partner who had self-tested—“The fact that I thought she had chlamydia really did put me off her”. In these cases, self-testing was seen to indicate a high likelihood the person was infected. On the other hand, self-testing could signify responsibility and care for health—“I’d probably feel more confident in them…they take care of themselves so I’d be, I’d feel more safe with them” (F18(ST)).

Generally, participants felt that friends would respond quite positively to self-testing—“they would think it’s responsible” (F5(ST)). However, when participants discussed others’ opinions more widely, it was noticeable that none anticipated a purely positive response. For many, anticipated responses concerned assumptions of unsafe sexual activity and promiscuity—e.g., “she’s had a chlamydia test she must be a slag” (F5(ST)). These fears may be well grounded. One participant received negative reactions on mentioning her self-testing to colleagues—“their reaction wasn’t positive, they just looked at me like why, like it was a bad thing to do” (F19(ST)). Such attitudes may limit access to self-testing, e.g., people might be deterred from buying a self-test at a pharmacy counter—“I’d think they were maybe judging me…I’d worry that they thought I was the sort of person that slept around a lot” (F18(ST)). Given the anticipated negative response to self-testing, it is not surprising that some participants would be careful about to whom they would reveal test kit use. Nevertheless, these issues may be relevant to chlamydia testing however the test might be performed; indeed, self-testing may be preferable to being seen in a clinic where contact with others is unavoidable. 

Self-testing in others (where the tester was not the individual’s partner) was generally perceived less negatively than participants thought others would perceive their own use of testing—“sensible and sort of cared about their health” (F19(ST)). Some recognised that self-testing could result from poor health behaviours, but felt that credit was due for now acting. Others took a population perspective on the health benefits—“I’d think great…the more people that know that they’ve got it the less it’s going to spread” (F17(ST)). F4(NT) viewed testing as a choice between self-testing and testing at a clinic, and was happier about her friends self-testing, anticipating visiting a clinic to be a highly embarrassing experience—“I’d prefer it that they use the…self kit rather than the actual going to the clinic”. However, these findings were from a group of people who volunteered to take part in a study about self-testing for chlamydia, so they may view self-testing as less stigmatizing than people who did not come forward to take part. Even so, some participants felt that self-testing could reflect negatively on the person—“Unless I could kind of see that they were doing it because they’re just being sensible, I’d probably just think that they were…promiscuous or they sleep with people who are” (M16(ST)). This latter participant had visited a doctor with concern about a chlamydia infection, and had also self-tested at the Students’ Union. For him to reluctantly admit that he would probably think negatively about people who self-tested demonstrates how deeply stigmatised STIs can be. Self-testing could also be seen as secretive, implying unfaithful behaviour—“they’re trying to hide something from their partner” (F1(NT)).

### 3.7. Relating Findings to Theoretical Models

Protection Motivation Theory (PMT, [15,16]) suggests that believing the behaviour will reduce the threat (response efficacy), believing that one can perform the behaviour (self-efficacy) and response costs will affect motivation to perform a behaviour—in this case, self-testing for chlamydia. Response efficacy seemed highly salient, when operationalized as believing the behaviour would lead to an accurate test result (after which people could take appropriate action to manage the infection). Participants concerned about test accuracy preferred to attend a clinic rather than to self-test. Self-efficacy was also important, with some lacking confidence in using a vulvo-vaginal swab. These concerns were linked with response efficacy: people worried that they could not self-sample correctly, leading to inaccurate results. Self-efficacy has predicted behaviour in a range of contexts, including notifying partners of STIs [21]. Perceived response costs to self-testing included financial costs of purchased tests and the risk of being discovered. However, costs were also associated with the alternative behaviour of being professionally tested, e.g., embarrassment and time costs. 

Using the Theory of Planned Behaviour (TPB, [17]), intention to self-test should be predicted by attitude towards self-testing, perceptions of whether others think one should perform the behaviour (subjective norm), and perceived behavioural control (similar to self-efficacy discussed above). Participants varied in their attitudes towards self-testing. Opinions seemed to largely depend on whether they thought results would be accurate and weighing up the costs and benefits of self-testing and professional testing. In this context, “attitude” seems highly related to the PMT domains of response efficacy and response costs. “Subjective norm” was of particular relevance. Participants could be encouraged to self-test if friends were positive about the behaviour, whereas the stigma surrounding STIs and implications of testing on relationships could limit self-testing. Alternatively the openness with which they would test could be limited, perhaps driving people towards secretly self-testing instead of attending clinics. This theoretical domain supports the importance of either normalising testing or of providing increased privacy.

PMT and TPB, therefore, have considerable relevance to self-testing for chlamydia. Participants often considered self-testing in comparison with testing by a professional, rather than as opposed to not testing at all. Future research would benefit from incorporating theoretical frameworks and taking account of this choice context when operationalizing theories.

## 4. Discussion

Reasons given for self-testing, and perceived benefits of self-testing, included its seeming ease, convenience, anonymity, and not requiring consultation or physical examination. Provision of incentives (t-shirts) and social acceptance (or privacy—not needing anyone to know) seemed to encourage testing; financial costs of self-testing were off-putting. These findings were largely consistent with research on chlamydia screening more generally [22,23]. An interview study of 14 young adults who declined chlamydia tests in non-medical settings found that embarrassment about being tested, and perceptions that a chlamydia test involved an uncomfortable physical examination, seemed to be factors in people declining chlamydia tests [24].

Test accuracy seemed the main concern for participants; tests taken by professionals were perceived as more reliable than self-tests. Lacking professional support when receiving a positive result was viewed as anxiety-provoking. However, participants reported that they would urgently seek help on receiving a positive self-test result. Anxiety triggered specifically by isolation may, therefore, be of short duration. When participants were asked how they would feel more generally about a chlamydia diagnosis (*i.e*., not specifically after self-testing), the range of negative emotions anticipated and the sense of urgency in obtaining treatment were very similar to those anticipated specifically after self-testing. Thus, these exploratory results suggest that the impact of a positive result might be similar, however it be delivered.

For female participants, using a vulvo-vaginal swab could be worrying in case sampling error reduced test accuracy. Studies of self-sampling for human papillomavirus using vaginal swabs indicated similar concerns for participants [25,26]. The option of urine sampling might encourage testing for chlamydia. Stephenson *et al.* (2000) sent female participants either a urine testing kit (n = 103) or a vulval swab kit (n = 105) (kit type was determined at random); a higher response rate that just reached statistical significance was seen in those sent urine kits (47% *vs* 32%, *p* = 0.05) [27]. Hsieh *et al.* (2003) asked women to provide both urine and vaginal swab samples before completing a questionnaire indicating their preference [28]. The vast majority of participants (90.8%) felt comfortable collecting a urine sample, compared with 69.6% feeling comfortable collecting vaginal swab samples (*p* < 0.001, n = 1382) [28]. Two studies conducted qualitative interviews with a subset of participant in large chlamydia screening studies [10,29]. Women who had used a vulvo-vaginal swab expressed “unease” with the experience [10] whereas in Pimenta *et al.*’s (2003) study, where urine samples only were requested, women found the process straightforward [29]. However, findings are not unanimous; Chernesky *et al.* (2005) found preference for self-collection with a vaginal swab over urine collection, although tests of statistical significance in this quantitative questionnaire study were not reported [30]. Little research has addressed why women might prefer one sampling method over the other. If this concern about sampling error is generalizable then an intervention improving self-efficacy may increase willingness to use vulvo-vaginal swabs. Interestingly, evidence suggests that women’s concern about sampling error using vulvo-vaginal swabs is unfounded. Self-sampled vulvo-vaginal swabs have been found to be significantly more sensitive than endocervical swabs taken by a clinician [31]. 

In the present study, participants who self-tested at the Students’ Union seemed to be particularly encouraged to self-test by the t-shirt incentives. Similarly, a cash incentive was found to be effective in encouraging students to be screened in an Australian study [32]. The social atmosphere at the Students’ Union also supported testing. Previous research has suggested that screening should be normalised within society [33,34]; the Students’ Union testing seems to have achieved this. In contexts where normalisation of testing is not feasible it could be helpful to maximise the privacy of self-testing, e.g., by providing kits in locations where no one will observe their collection.

This study extends previous findings into a new and important context: self-testing for chlamydia outside of formal medical or screening contexts. Of particular importance is the use of theory. Relating responses to theoretical models allows us to understand our findings in the context of wider behaviour-related research and to create frameworks for further larger-scale, quantitative research and for potential public health interventions. 

The study was restricted to a well-educated young adult sample. However, this age group is important in this context because young adults (aged 16 to 24 years) are the group at highest risk of contracting sexually-transmitted infections [13]. An important advantage of this sample is that, unlike other studies, we recruited people outside screening programmes or medical settings to identify issues relevant to self-test use in the “real world”. 

No participant in the present study received a positive test result, and they accepted invitations to test rather than actively seeking a self-test kit. This was despite widespread advertising of the study to students and a £20 incentive to take part. It may be that the stigma surrounding STIs is such that few people with a positive result are willing to discuss it, and that people who seek the privacy of self-testing are unwilling to participate in face-to-face interviews. Nevertheless, it is important to recruit such individuals to research to address the impact of a positive test result. A much larger-scale, multi-site recruitment process may have identified individuals who would have been willing to be interviewed, but such a process was beyond the resources of the present study. In addition, if so few people in this group are willing to be interviewed face-to-face, it would seem likely that those who are willing could differ in important characteristics to more private individuals. A more constructive approach might be for future studies to use methodologies that allow greater concealment of identity to facilitate this recruitment. We have recently successfully used on-line advertising and on-line questionnaires to ask people about self-testing in a quantitative study [35]. It may be that online methods of both advertising studies and interviewing participants may make it easier for private people with stigmatized conditions to take part, with a higher degree of anonymity. Nevertheless, the present study explored how participants felt they would respond, were they to receive a positive self-test chlamydia result, so we were able to consider decision-making in members of this at-risk population.

Finally, we had a gender imbalance in our sample, with only three of our 18 participants identifying as male. We are uncertain why male students were less likely to come forward to take part. It may be that men were less willing to talk about their health, especially about a sexual activity-related condition, when it was clear from adverts that the lead researcher was female. We did not purposively sample by gender in the present study, but future researchers should consider doing so. Recruitment procedures could be developed to specifically target male groups, and allowing participants to choose the gender of the interviewer might facilitate the participation of men. 

## 5. Conclusions

This study suggests that self-testing for chlamydia is perceived to be acceptable, and has identified factors that may be useful in predicting and increasing self-testing behaviour, including response efficacy, self-efficacy, social influence/privacy, and incentives. The relevance of theory has been demonstrated; using theoretical frameworks will facilitate the effective structuring of future studies and interventions aimed at increasing self-testing for chlamydia. The qualitative findings, placed within theoretical models, suggest that interventions that increase women’s confidence in using self-tests (especially vulvo-vaginal swabs) and reassurance that tests provide accurate results may increase people’s intentions to self-test. Increasing the social acceptability and desirability of self-testing, and reducing stigma related to testing for sexually transmitted infections may also be beneficial. However, research needs to ascertain negative impact in people who receive positive self-test results, and to test whether the present study’s findings can be replicated in larger, more generalizable samples.

## Figures and Tables

**Table 1:** Description of participants.

Characteristic	Number (n) or Median (Range)
**Median age (years)**	22.5 (18–26)
**Ethnicity (n)**	
White British	8
Pakistani British	1
Indian British	2
Canadian (various ethnicities)	4
**Gender (n)**	
Female	15
Male	3
**Median number of lifetime sexual partners**	3.5 (1–8)
**Median number of new sexual partners (in last 12 months)**	0 (0–4)
**Previous self-test use (n)**	
Had self-tested	11
Had not self-tested	7
**Previous self-test location (n)**	
Home	3
University Students’ Union	5
Clinic	3

Note: n = number of participants.

## Abbreviations

The following abbreviations are used in this manuscript:

STI	Sexually transmitted infection
PMT	Protection Motivation Theory
TPB	Theory of Planned Behaviour
